# Precision excision of mandibular anterior compound odontoma using autonomous robotic guidance: a clinical case report

**DOI:** 10.3389/froh.2025.1661277

**Published:** 2025-09-10

**Authors:** Tiankai Di, Chen Liu, Yuhan Liu, Shizhu Bai, Li-an Wu, Yujiang Chen, Yimin Zhao

**Affiliations:** 1 State Key Laboratory of Oral & Maxillofacial Reconstruction and Regeneration, National Clinical Research Center for Oral Diseases, Shaanxi Clinical Research Center for Oral Diseases, Department of Pediatric Dentistry, School of Stomatology, The Fourth Military Medical University, Xi’an, Shaanxi, China; 2 State Key Laboratory of Oral & Maxillofacial Reconstruction and Regeneration, National Clinical Research Center for Oral Diseases, Shaanxi Clinical Research Center for Oral Diseases, Department of Digital Dental Center, School of Stomatology, The Fourth Military Medical University, Xi’an, China; 3 Department of Neurobiology and Institute of Neurosciences, School of Basic Medicine, Fourth Military Medical University, Xi’an, Shaanxi, China

**Keywords:** compound odontoma, robotic surgery, autonomous guidance, minimally invasive surgery, mandibular anterior region, case report

## Abstract

**Background:**

Management of compound odontomas in the pediatric anterior mandible poses significant surgical challenges due to proximity to developing tooth follicles and neurovascular structures. Conventional enucleation risks iatrogenic injury to adjacent dentoalveolar anatomy, while suboptimal bone preservation may impede permanent tooth eruption.

**Case description:**

An 8-year-old patient presented with a compound odontoma adjacent to the unerupted permanent mandibular incisor. Utilizing an autonomous robotic guidance system independently developed by our research group, we performed minimally invasive enucleation featuring: (1) virtual osteotomy pathway planning, (2) sub-millimeter precision bone removal preserving the follicular space of tooth 31, and (3) capsule dissection under optical navigation. At the 2-week follow-up, the surgical site demonstrated complete mucosal healing without neurosensory complications, and CBCT confirmed absence of residual pathology.

**Conclusion:**

Robotic-assisted enucleation enabled tissue-preserving removal of a high-risk odontoma while maintaining eruption potential. This approach represents a paradigm shift toward precision-targeted dentoalveoral surgery, particularly valuable for anatomically complex pediatric cases.

**Clinical Trial Registration:**

## Background

Odontomas are the most common odontogenic tumors (1–4). Odontomas had a marked predilection for the mandible and for the anterior region of the jaws, particularly for the anterior maxilla (1). These benign lesions often impede permanent tooth eruption and cause malocclusion, particularly in pediatric and adolescent patients (5). Conventional surgical enucleation—while effective—carries risks of iatrogenic damage to adjacent dental structures, neurosensory impairment, and prolonged healing in anatomically complex regions like the anterior mandible (6–8).

Recent advancements in robotic-assisted surgery have demonstrated significant potential for improving procedural accuracy in oral and maxillofacial procedures (9, 10). Contemporary autonomous robotic systems achieve submillimeter precision in osteotomy execution, provide real-time three-dimensional navigation, and facilitate minimally invasive approaches, thereby potentially minimizing iatrogenic tissue damage (11, 12). However, the current evidence base remains insufficient to fully evaluate the efficacy of robotic systems for complex dentoalveolar pathology management, particularly in cases requiring odontoma resection. This evidence gap is most pronounced regarding longitudinal outcomes and pediatric applications (13, 14).

This report details a case involving a pediatric patient diagnosed with a compound odontoma situated adjacent to the unerupted mandibular permanent incisor (#31). The lesion was managed via autonomous robotic enucleation employing an autonomous robotic guidance system developed in-house by our research group. The procedure integrated cone-beam computed tomography (CBCT)-based preoperative planning, dynamic surgical pathway mapping, and tissue-sparing osteotomy. Primary emphases encompassed: (1) optimization of minimal bone removal to facilitate eruption potential, (2) validation of robotic precision through postoperative CBCT assessment, and (3) evaluation of early-stage healing responses. Our objectives were to demonstrate the feasibility of robotic surgery for complex dentoalveolar pathologies and to catalyze discourse on technological synergies in precision-based oral healthcare.

## Case description

1 Initial Examination:
  1.1 Chief complaints:

The patient requested enucleation of the odontoma in the anterior mandibular region.

1.2 Medical History:

The patient has no significant medical history.

1.3 History of present illness:

The patient, an 8-year-old boy, was referred to our hospital with a request for odontoma enucleation, which had been identified in the anterior mandibular region during an examination at another institution one month prior.

1.4 History and Habits

The patient has no significant history and habits.

1.5 Extraoral Examination

Extraoral examination showed no significant abnormalities.

1.6 Intraoral Examination

**Figure 1:**
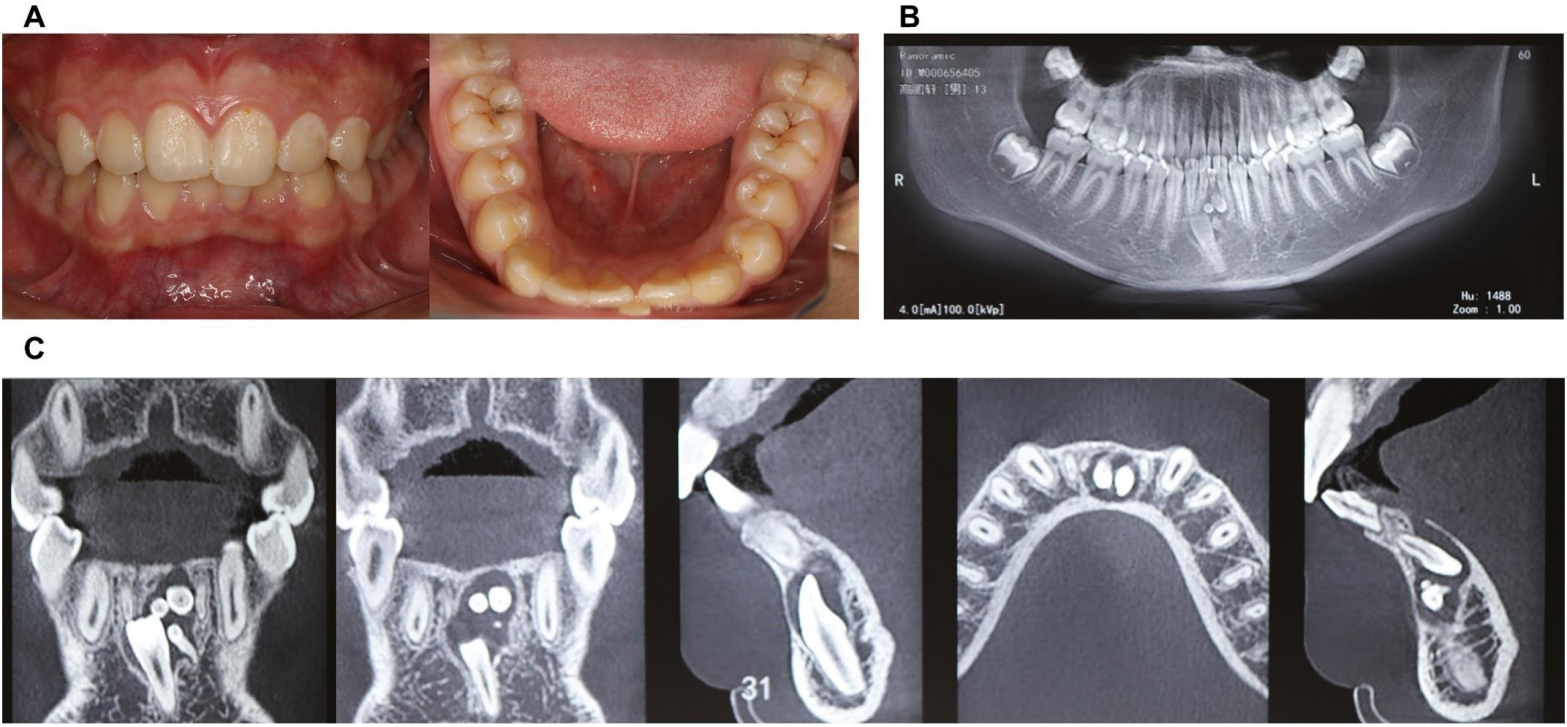
Physical and CBCT examination of compound odontoma. **(A)** Photos of intraoral view; **(B)** CBCT examination (overall view); **(C)** CBCT examination (location of compound odontoma in the anterior mandibular region, including sagittal, coronal, and axial planes).

Intraoral examination revealed retained primary tooth 71 and unerupted permanent tooth 31 (Figure 1A). CBCT imaging identified a compound odontoma adjacent to tooth 31 (Figures 1B,C). After discussing the treatment plan, surgical procedure, and associated risks/benefits with written instructions, the guardians consented to autonomous robotic extraction of the compound odontoma (Yakebot; Yakebot Technology Co., Ltd., Beijing, China).

2 Diagnosis and Prognosis

31 teeth was completely bone-impacted, with compound odontoma adjacent to tooth 31.

**Figure 2:**
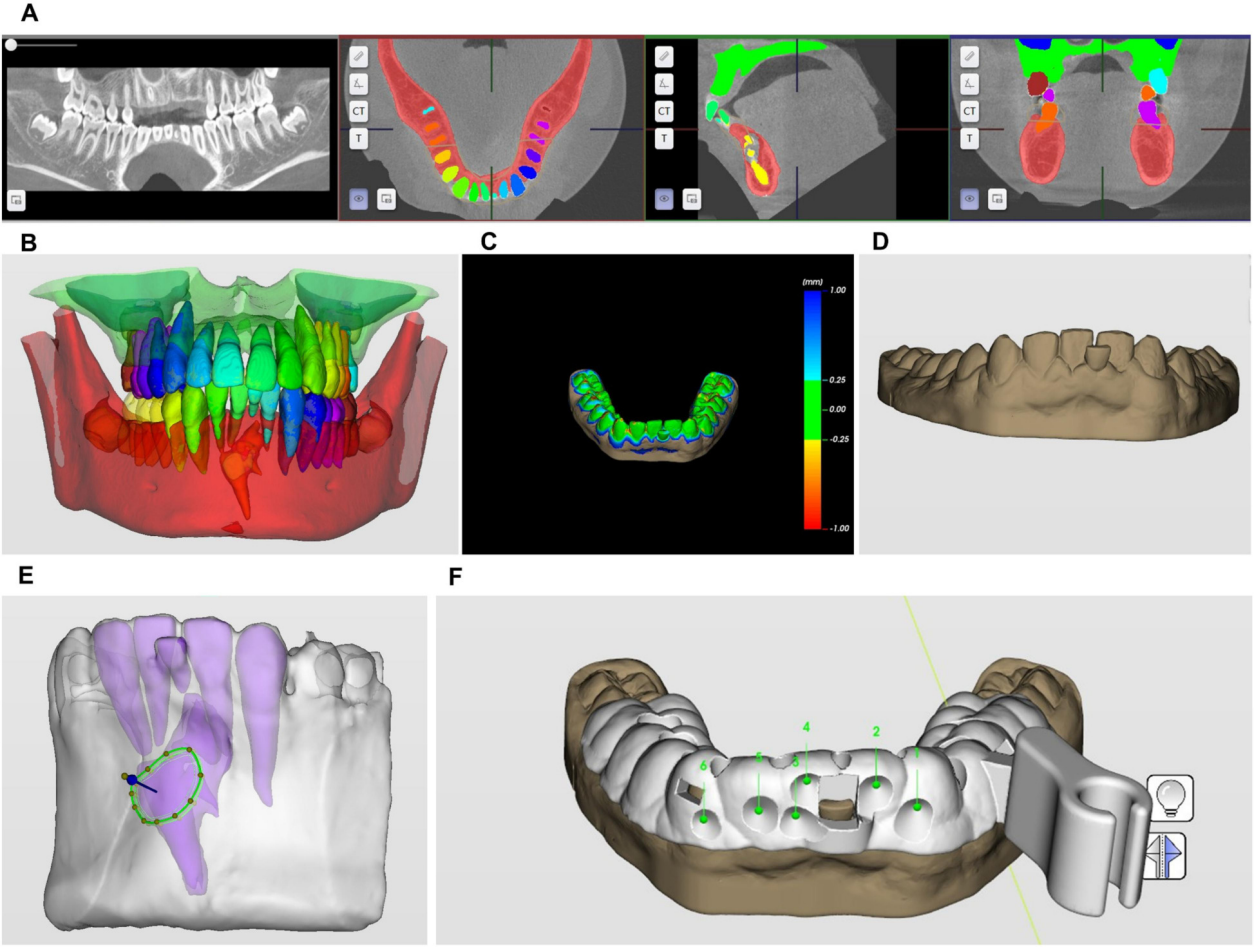
Three-dimensional robotic surgical planning workflow for compound odontoma excision. **(A,B)** CBCT data segmentation; **(C,D)** intraoral scan data matching; **(E)** minimally invasive bone removal and odontoma segmentation pathway planning; **(F)** surgical guide template design.

3 Intervention-Robot-assisted surgical planning design:
  3.1 CBCT data segmentation (Figures 2A,B);
  3.2 Intraoral scan data matching.

For robotic surgical planning, intraoral scans of the labial vestibule, teeth #36–46, and palatal mucosa were acquired using an intraoral scanner (CS 3600; Carestream Dental) and exported in STL format. Concurrently, CBCT data captured in DICOM format were processed through HiRes3D software (LargeV Instrument Corp.) to delineate the odontoma's spatial relationship with adjacent anatomy. These datasets underwent fusion to generate a 3D surgical field model for robotic navigation design (Figures 2C,D).

3.3 Minimally invasive bone removal and odontoma segmentation pathway planning.

Preoperative planning utilized Yakebot software (DentalNavi 3.0.0) to fuse CBCT-DICOM and intraoral-STL data for 3D reconstruction. The AI segmentation module precisely identified the compound odontoma and adjacent structures. Based on lesion topography and a 1.2 mm fissure bur, the system generated a minimal-access osteotomy pathway. A 3D-printed surgical attachment (SprintRay Pro S95) with registration holes and nerve-protective baffle was implemented intraoperatively. The baffle's 5 mm notch secured mucoperiosteal flap retraction without compressing neurovascular bundles, enabling stable robotic guidance during enucleation (Figure 2E).

3.4 Surgical guide template design (Figure 2F).

**Figure 3:**
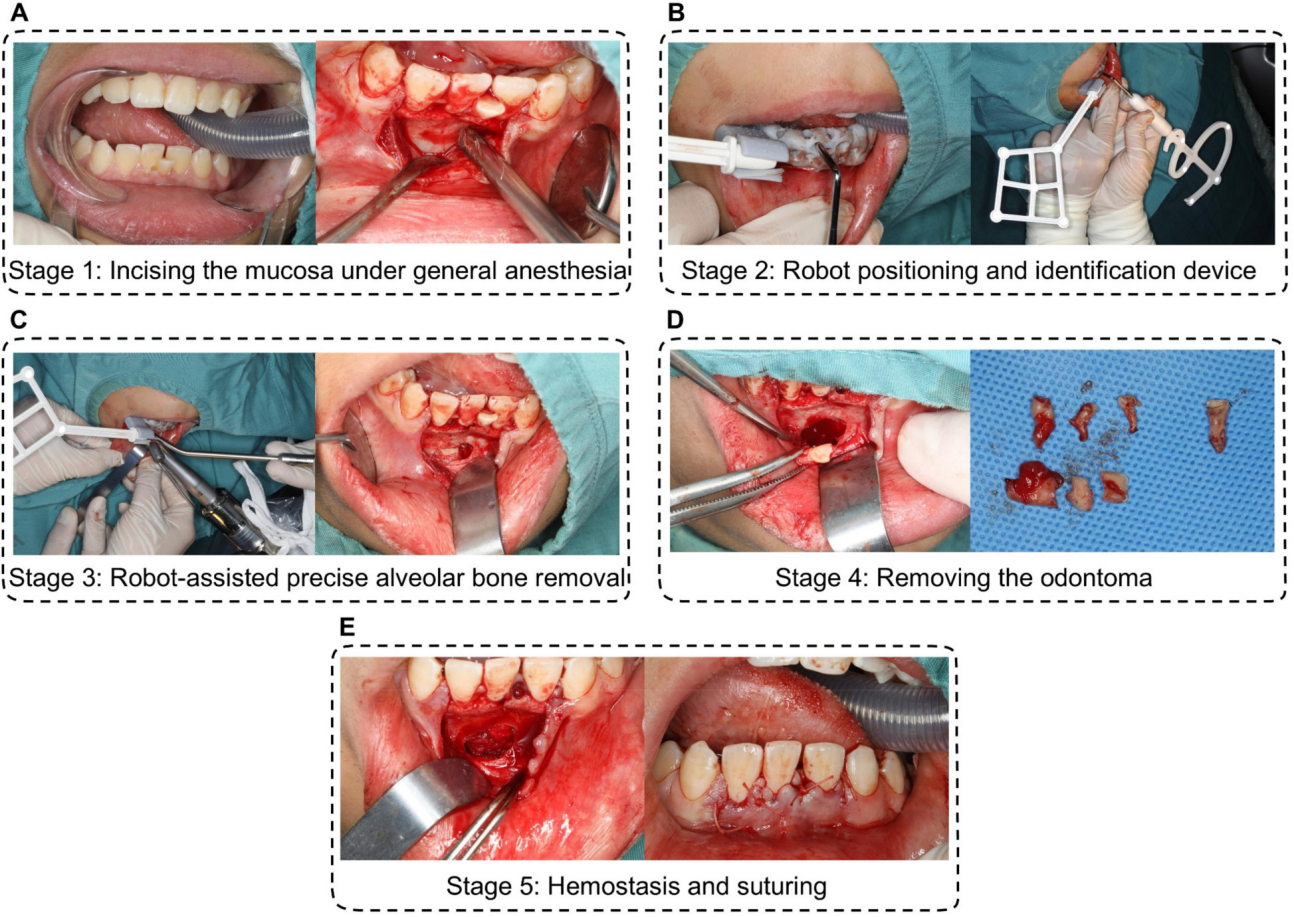
Procedure of precision excision of compound odontoma using autonomous robotic guidance. **(A)** incising the mucosa under general anesthesia; **(B)** robot positioning and identification device. **(C)** robot-assisted precise alveolar bone removal; **(D)** removing the compound odontoma and the impacted 31 tooth; **(E)** hemostasis and suturing.

4 Intervention-precision excision of compound odontoma using autonomous robotic Guidance:
  4.1 Incising the mucosa under general anesthesia;

Following preoperative disinfection with iodophor and local anesthesia (4% articaine hydrochloride with epinephrine), a incision was extended around tooth 31. Blunt dissection elevated a mucoperiosteal flap, exposing the underlying bone (Figure 3A).

4.2 Robot positioning and identification device.

The robotic arm was manually positioned near the surgical field. A custom 3D-printed accessory, equipped with optical markers and registration holes, was secured intraorally using dentition support. Spatial registration was achieved by probing five fiducial markers, establishing real-time navigation via infrared tracking between the robotic instrument and the surgical site (Figure 3B).

4.3 Robot-assisted precise alveolar bone removal.

A NSK handpiece mounted on the robotic arm performed osteotomy along a preplanned pathway. Surgeons initiated bone milling via foot-pedal control, with real-time progress visualized on navigation displays. Saline irrigation maintained thermal safety. The system autonomously halted drilling at predefined endpoints and retracted upon command, completing osteotomy within 5 min without damaging adjacent roots or neurovasculature (Figure 3C).

4.4 Removing the odontoma.

Following bone window creation, the compound odontoma and the impected 31 tooth were extracted. Continuous optical navigation prevented iatrogenic contact a the preserved neurovascular bundle, ensuring complete capsule removal (Figure 3D).

4.5 Hemostasis and suturing.

Following bone window creation, the compound odontoma was extracted intact using cupped elevators. Continuous optical navigation prevented iatrogenic contact with the preserved neurovascular bundle and unerupted tooth #31, ensuring complete capsule removal (Figure 3E).

**Figure 4:**
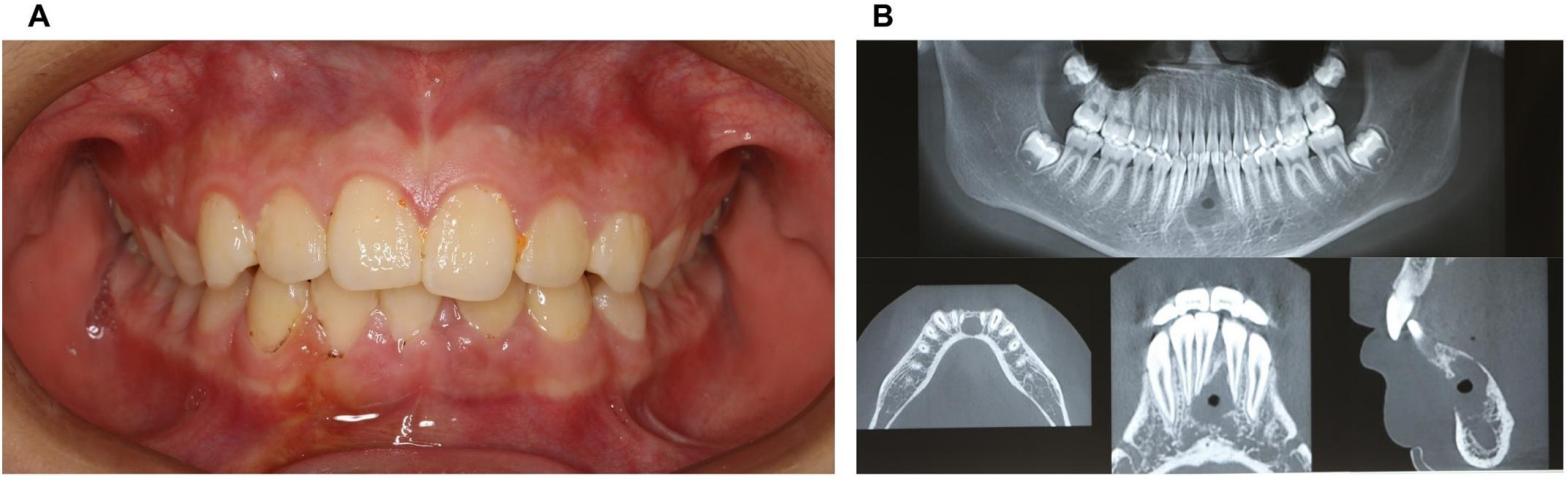
Physical and CBCT examination two weeks after surgery. **(A)** Photos of intraoral view; **(B)** CBCT examination.

**Figure 5:**
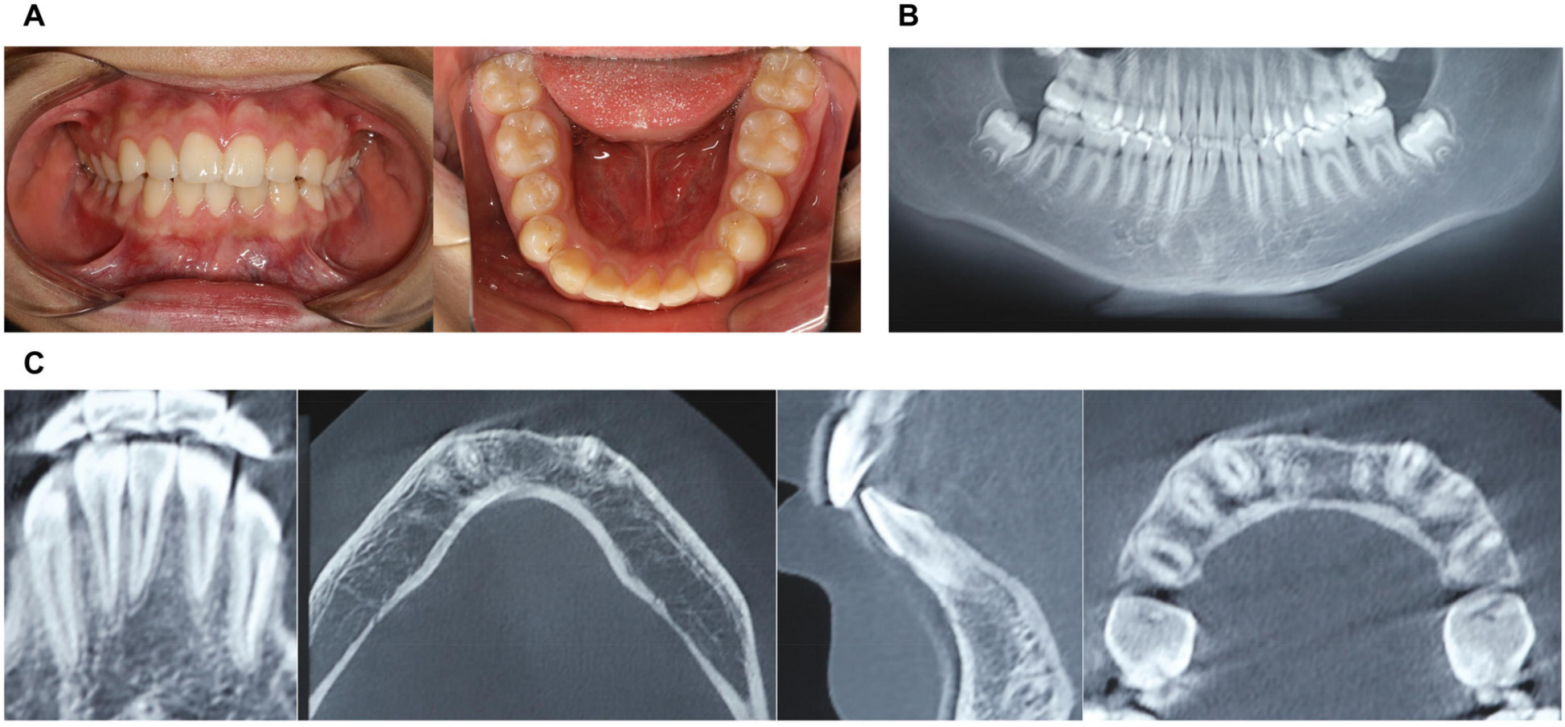
Physical and CBCT examination one year after surgery. **(A)** Photos of intraoral view; **(B)** CBCT examination (overall view); **(C)** CBCT examination.

5 Outcome-Follow-up of 2-week and 1-year after surgery:

The patient returned for a postoperative review two weeks after surgery, with the surgical site showing good healing and no reported discomfort (Figures 4A,B; Figures 5A–C).

## Discussion

Surgical management of compound odontomas in the anterior mandibular region remains clinically challenging due to intricate anatomical constraints (1–4). Conventional enucleation techniques, reliant on surgeon dexterity and spatial perception, frequently encounter difficulties in achieving complete lesion removal while preserving adjacent critical structures. Risks of iatrogenic injury to developing tooth follicles, neurovascular bundles, and thin buccal cortices are well-documented concerns (6–8). In contrast, robotic-assisted systems overcome these limitations through three-dimensional visualization and sub-millimeter motion control. This case demonstrates how preoperative CBCT data integration enables precise osteotomy pathway planning around the odontoma's periphery—effectively minimizing collateral tissue disruption while ensuring complete encapsulation removal—a critical advantage in anatomically complex pediatric cases (9, 10).

The paradigm of minimally invasive surgery is fundamentally redefined through robotic precision (15, 16). Traditional approaches often necessitate extensive bone removal and mucoperiosteal reflection to gain adequate operational visibility, resulting in prolonged healing and compromised functional outcomes. Robotic systems, however, allow for lesion-specific targeted osteotomy without compromising structural integrity. This case exemplifies significant soft tissue preservation with minimal incision lengths and negligible periosteal elevation. Postoperative assessment revealed accelerated mucosal healing compared to conventional benchmarks, with no neurosensory complications. Such tissue-sparing capabilities align optimally with the pathological characteristics of encapsulated odontomas, establishing a new standard for conservative dentoalveolar surgery.

Recent digital advances have popularized surgical guides and navigation systems for impacted tooth removal, demonstrating significant advantages in precise localization (17, 18). However, while these technologies markedly reduce localization time, they remain unable to facilitate complete tooth disimpaction (19). Substantial surgical effort is still required to overcome osseous and dental resistance along the extraction path. Our conventional surgery cohort data revealed that this disimpaction phase exceeded localization time and predominated in overall procedure duration. This persistent limitation may increase surgical trauma and prolong operative time even with navigational assistance, ultimately compromising procedural precision.

Our self-developed robotic system fundamentally advances compound odontoma management by resolving the critical limitation of current digital approaches (20)—while traditional surgical guides and navigation systems excel at lesion localization, they fail to achieve the aim of conservative osteotomy which constitutes the most time-consuming and technically challenging aspect of conventional surgery. The robotic solution integrates three paradigm-shifting capabilities: (1) Preoperative virtual resistance modeling that algorithmically calculates all bone interference zones along the optimal enucleation path, enabling ultra-conservative osteotomy planning tailored to the lesion's 3D morphology; (2) Autonomous execution via a specialized high-speed end-effector performing complex bone removal in anatomically confined spaces inaccessible to conventional instruments, dramatically accelerating the bone-resistance elimination process; (3) Quantitative surgical efficacy evaluation through osteotomy volume-to-odontoma volume ratios, eliminating subjective surgical field assessments that overlook lesion size variability.

Contrary to navigated surgery, which primarily offers spatial guidance without direct intervention, our autonomous robotic system achieves a high degree of procedural autonomy during critical phases. Specifically, the robotic arm autonomously executes the preplanned osteotomy pathway—including bone milling with a 1.2 mm fissure bur—under real-time optical navigation supervision. This is exemplified by its ability to autonomously halt drilling at predefined endpoints and retract upon command, as demonstrated in the bone removal phase where it completed osteotomy within 5 min without manual instrument manipulation. Such autonomy reduces human error and tremor, while quantitative postoperative CBCT analysis confirmed submillimeter accuracy, distinguishing it from passive navigation tools that still require surgeon-controlled execution.

This integrated approach significantly reduces iatrogenic risks to developing follicles and neurovascular bundles while preserving maximal bone stock—advantages objectively verified by postoperative volumetric analyses showing substantially minimized tissue sacrifice. Crucially, the system allows surgeons to oversee continuous, tremor-free milling along prevalidated paths through simple foot-pedal control rather than manual instrument manipulation. This transforms odontoma enucleation from a technique-sensitive, experience-dependent procedure into a reproducible precision intervention where the robot executes the physiologically taxing bone-removal phase with submillimeter fidelity. The resultant reduction in operative duration, operator fatigue, and anatomical disruption establishes a new gold standard for managing deeply impacted odontomas in pediatric patients. Our autonomous robotic system has undergone rigorous validation through the registered clinical trial where we established a standardized clinical pathway encompassing: (1) CBCT/intraoral scan fusion for virtual planning, (2) optical-navigated robotic execution demonstrated in our surgical workflow, and (3) quantitative outcome assessment protocols. Based on current regulatory review progres, the robotic surgical system is projected for expanded clinical deployment, with phased implementation across multiple medical institutions.

## Conclusion

This case substantiates three core advantages of robotic-assisted enucleation: (1) Critical Structure Safeguarding through tremor filtration and motion constraint algorithms preventing incidental contact with follicles and nerves; (2) Programmatic Precision Execution translating virtual plans into physical operations with sub-millimeter fidelity; and (3) Predictability Enhancement verified by postoperative CBCT confirmation of complete lesion removal and bone preservation. The integration of artificial intelligence heralds transformative potential—next-generation systems could utilize real-time optical navigation feedback to dynamically adjust osteotomy paths during drilling, while predictive algorithms may forecast eruption probabilities based on follicular biomechanics. Such synergistic “precision intelligence” platforms promise to transition odontoma management from reactive intervention to biologically optimized preservation therapy.

## Data Availability

The original contributions presented in the study are included in the article/Supplementary Material, further inquiries can be directed to the corresponding authors.
